# Observation of Kelvin–Helmholtz billows in the marine atmospheric boundary layer by a ship-borne Doppler wind lidar

**DOI:** 10.1038/s41598-025-89554-4

**Published:** 2025-02-12

**Authors:** Shokoufeh Malekmohammadi, Etienne Cheynet, Joachim Reuder

**Affiliations:** 1 Geophysical Institute, and Bergen Offshore Wind Centre, University of Bergen, Allegaten 70, 5007 Bergen, Norway; 2 Bjerknes Centre for Climate Research, Bergen, Norway

**Keywords:** Kelvin–Helmholtz instability, Marine atmospheric boundary layer, Doppler wind lidar, Turbulence, Fluid dynamics, Wind energy, Atmospheric dynamics

## Abstract

Kelvin–Helmholtz billows (KHB) and the associated turbulence characteristics in the atmospheric boundary layer (ABL) are mainly investigated through simulations and limited observations. Traditional methods using in-situ wind sensors are constrained by mast height, resulting in a limited understanding of KHBs at higher altitudes. Lidar remote sensing provides a promising approach for studying KHBs at altitudes above 100 m. This study presents observations of KHBs in the marine ABL above 600 m, through ship-borne lidar observations. Two Doppler wind lidars, one scanning lidar, and one wind profiler, were installed for several months on a crew transfer vessel, operating in the Rødsand 2 wind farm off the coast of Denmark. On 2023-02-22, KHBs were detected between 600 and 800 m altitude over 10 min. The standard deviation of vertical turbulence was found to increase by a factor of two during KHB occurrence. The power spectral density of vertical fluctuations showed a greater increase in the frequency range below 0.1 Hz, with a peak indicating a periodic pattern with a period of 55 s. The kurtosis of the vertical component also showed a large increase near the edge of the billows, as documented in the scientific literature for billows occurring near the surface. The billows triggered a downward mixing of aerosols and momentum to around 550 m. Although no interaction between the wind farm and the KHBs was observed, we hypothesise that KHB may reduce wind farm wake losses in shallower stable layers above the farm by enhancing vertical mixing and downward momentum transport.

## Introduction

Kelvin–Helmholtz billows (KHBs) result from an instability in hydrostatically stable conditions, that occurs at the density interface when vertical shear exceeds a critical level and the flow becomes dynamically unstable^1–3^. They manifest as waves or “billows”, aligned with the shear stress. Starting as small wave disturbances at the density interface, their amplitude rapidly increases with time, causing them to roll up, overturn, and finally break. By that, Kelvin–Helmholtz instability can contribute to considerable turbulent mixing in the vertical direction in otherwise stable atmospheric conditions. In wind energy meteorology, such an increased mixing might be important by potentially providing a mechanism for accelerating the recovery of the wakes created by turbines or wind farms.

As the envisaged green energy transition, required to reduce anthropogenic CO_2_ emissions and limit the effects of climate change^4,^ calls for a considerable upscaling of offshore wind energy^5,6^, the density of offshore wind farms is expected to increase in certain areas, such as the North Sea. Therefore, a better understanding of the physics of the atmospheric flow within and around wind farms is needed^7^. Veers et al. in ‘Grand Challenges in the Science of Wind Energy’^7^ emphasise the need for novel and improved measurement systems and observational strategies to better understand the undisturbed atmospheric flow and wind farm interactions.

In this context, an experiment near the Rødsand 2 wind farm off the coast of Lolland, Denmark, utilised ship-borne Doppler wind lidars to investigate atmospheric dynamics and their interaction with wind turbines within the farm. The campaign specifically aimed to characterise the vertical structure of the atmospheric flow within and surrounding an offshore wind farm, extending from the surface to the top of the atmospheric boundary layer (ABL)^8^. This paper highlights the potential of the measurement setup using a unique case study. On 2023-02-22, the ship-borne scanning wind lidar in vertical stare mode captured KHBs at heights between 600 m and 800 m above the wind farm. Although the KHBs remained well above the wind farm, without interacting with its wake, their ability to enhance vertical mixing is valuable for boundary layer meteorology and wind energy science. High-resolution observations of such events remain uncommon, yet are essential for advancing knowledge in these fields.

Our knowledge of KHBs in the ABL originates mainly from numerical simulations^9–11^ and limited atmospheric studies^12–16^, with particularly sparse observations over the sea^17,18^. Observations of KHBs in the lower ABL close to the surface are typically based on 3D wind measurements by sonic anemometers on meteorological masts and towers,^12,13,15,16^, often complemented by tethersonde systems^12,15^, as well as lidar^13,15^, radar^15^ and sodar^12,15^ remote sensing. They are typically associated with distinct wind shear layers, e.g., low-level jets (LLJ) on the top of shallow stable boundary layers^13,14,18^. Some KHB observations are also reported at the top of the evolving shallow convective layer during the morning transition of the boundary layer for example at Dome C in Antarctica^19^ and in the flow over a forest canopy^20^. Higher up in the ABL, KHBs can be triggered by elevated LLJs^21^, land-sea breeze circulations^16^, and wind shear on top of the residual layer^22–24^. KHB events in the lower ABL are typically short-lived (< 30 min) and have a typical spatial dimension of a few hundred metres. The corresponding temporal scales are in the order of tens of seconds to a few minutes^13,19^. At higher ABL levels, the spatial scale of KHBs can increase to several kilometres and persistent shear can considerably increase their lifetime^22,25^.

KHBs generate turbulence that can, in principle, be studied using traditional in-situ wind sensors such as sonic anemometers on met-masts^13^. However, the height of the mast or tower restricts this method to KHBs occurring at rather low altitudes, typically below a few hundred metres^13,19^. The limitations of mast-based measurements stem from their fixed observation point. Airborne wind sensors can extend both the horizontal and vertical range for corresponding observations^17^. For KHBs at higher levels in the ABL, lidar remote sensing provides the most promising approach^13,15,23^. Medium- and Long-range scanning Doppler wind lidars can measure the line-of-sight (LOS) velocities over a range of several kilometres with a constant spatial resolution in the order of 25 m and a temporal resolution of 1 s. With such instruments, various observational strategies can be employed for the detection of KHBs. One possibility is to perform range height indicator (RHI) scans, i.e. a scan with varying elevation angles along a fixed azimuth direction, preferably oriented in line with the direction of the shear vector^13,14^. This results in a snapshot of the KHB during the duration of the scan, typically lasting a few tens of seconds. The spatial resolution of the observations is, in this case, determined by the selected range gate length, the lidar is operating with, typically 25, 50 or 100 m. Another measurement strategy is to operate the lidar in vertical stare mode while the KHB is advected through the lidar beam. One example is the study by Das et al.^23^ that combines the vertical beam measurement during lidar operation in Doppler Beam Swinging (DBS) mode for wind profiling, providing one direct vertical velocity measurement along the lidar beam about every 5 s. The spatial resolution of the KHB structures in this setup was mainly determined by the speed at which such structures were advected over the lidar position and the repetition rate of the vertical beam measurements.

In this study, we present one of the first high-resolution observations of KHBs in the marine atmospheric boundary layer, using a ship-borne Doppler wind lidar in continuous vertical stare mode. This setup enabled unprecedented spatio-temporal resolution, with vertical wind velocity measured at a temporal resolution of 1 s, which is up to five times faster than in previous studies^23^. In addition to vertical velocity measurements, we used the carrier-to-noise ratio (CNR) from the lidar as a proxy for aerosol distribution, providing a valuable direct visualisation of KHB structures. These high-resolution data offer new insights into the formation, development, and turbulence characteristics of KHBs. Our findings demonstrate the potential of advanced remote sensing technology to quantify non-stationary vertical turbulence in the upper ABL due to KHBs.

The paper is organized as follows: the instrumentation and methods section details the measurement setup employed during the observation of the KHB, elaborating on the placement of the scanning and profiling Doppler wind lidar within the offshore wind farm. The results section presents the observed KHBs, focusing on flow characteristics and time-frequency analysis of the phenomenon. This section also discusses the atmospheric conditions preceding and following the KHBs, utilising in-situ measurements and hindcast data. It further quantifies the turbulence increase resulting from the formation of the KHBs. This research contributes to both fundamental atmospheric science and wind energy science. It is particularly relevant for airborne wind energy systems operating between 200 and 800 metres, where KHB-induced turbulence may influence wind loading^26^. In addition, for traditional bottom-fixed wind turbines, KHBs occurring at lower altitudes may provide an additional potential effect to accelerate wake recovery in large offshore wind farms.

## Instrumentation

**Fig. 1:**
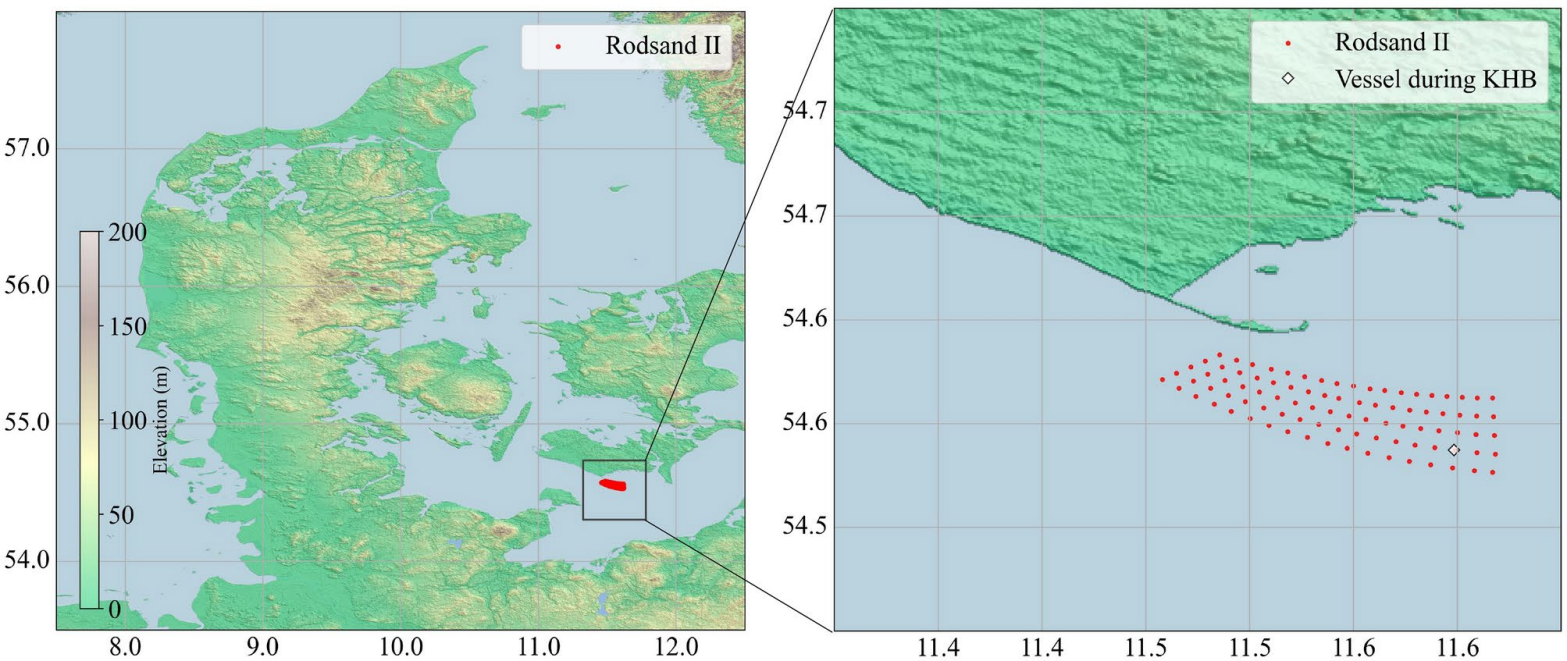
Vessel location near Lolland (Denmark) at the time of the observation of the Kelvin–Helmholtz billows. At the time of the observation, the vessel was stationary behind a wind turbine and 4 km away from the nearest shore. This map was created using Rasterio (an open-source Python library by MapBox) version 1.4.2 (https://rasterio.readthedocs.io/). The digital elevation model was obtained from SRTM data V4, provided by the International Centre for Tropical Agriculture (CIAT)^28^, available at https://srtm.csi.cgiar.org.

The Lollex measurement campaign, conducted from September 2022 to August 2023, was a collaborative effort between Train2Wind and the RWE group. Train2Wind is a European training network funded by the EU’s Horizon 2020 program under the MSCA-ITN scheme, dedicated to advancing the understanding of entrainment processes in large offshore wind farms. The campaign took place in the area of the Rødsand 2 wind farm (Denmark) operated by RWE group^27^. This wind farm, commissioned in 2010, consists of 90 Siemens turbines with a nominal capacity of 2.3 MW, a hub height of 68.5 m, and a rotor diameter of 93 m^27^ (see Fig. 1).

**Fig. 2:**
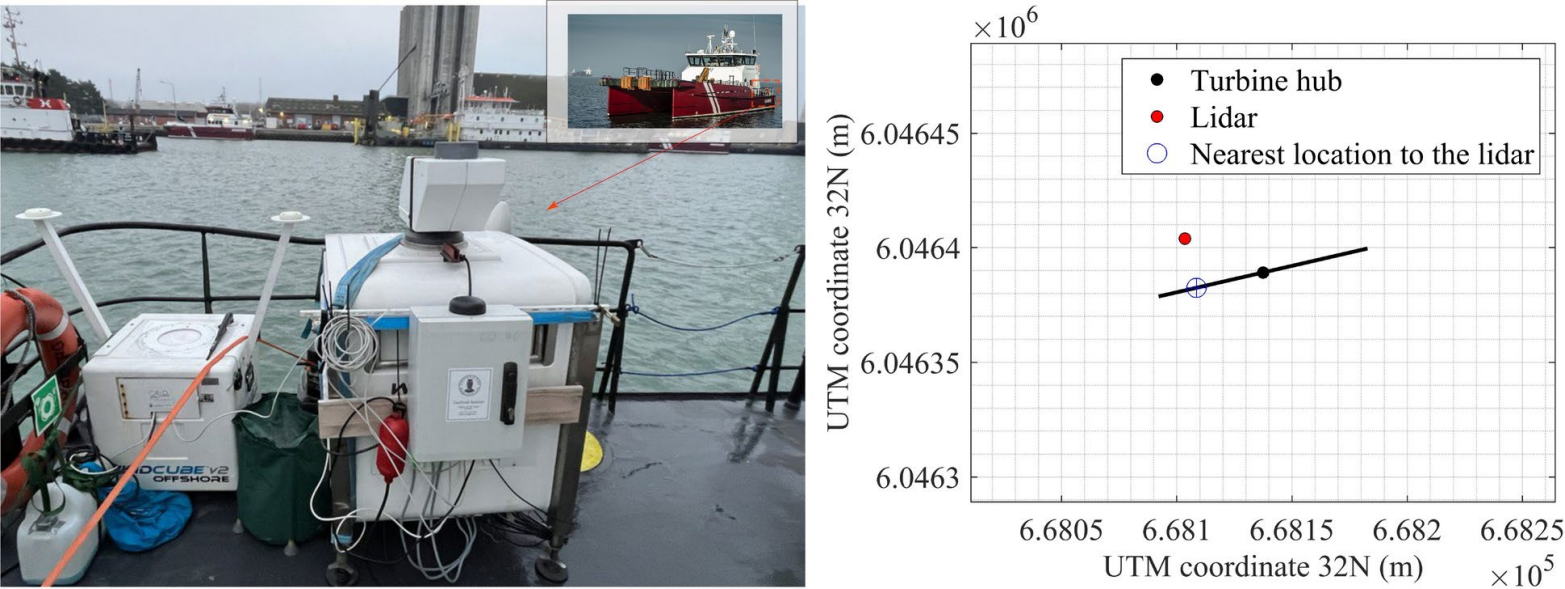
Left: Placement of lidars on the CTV: WindCube V2 lidar wind profiler (left) and WindCube 100S scanning lidar (right) located on the Stern. Right: Location of the ship-borne lidar in the vicinity of the nearest wind turbine inside the wind farm. The lidar is 37 m on the northwest of the hub and in the near wake zone of the wind turbine. The distance between the beam and the nearest point on the rotor is ca. 22 m.

Two Doppler wind lidars, installed onboard a crew transfer vessel (CTV) as an observational platform of opportunity, make a main instrumental contribution to the Lollex campaign. Doppler wind lidars are remote sensing instruments that record aerosol motion using infrared lasers to estimate the wind speed. The first lidar, a Leosphere WindCube V2 offshore wind profiler (Fig. 2 left), measures the wind speed and direction at heights from 40 m to 300 m above the surface with a vertical resolution of 20 m and a sampling frequency of 0.25 Hz. This instrument is equipped with an inertial measurement unit (IMU), that provides precise information on the location, orientation, and motion of the lidar with a temporal resolution of 10 Hz. This allows for the application of both, internal and external motion compensation algorithms for wind profiling^29,30^. The WindCube V2 lidar was also validated for wind profiling on buoys in Gottschall et al. 2014^31^. For a broader context, a review of floating lidar technology can be found in Gottschall et al. 2017^32^. The second lidar, a Leosphere WindCube 100S (Fig. 2 right), is a scanning lidar capable of high-resolution hemispherical scanning, with 1 Hz sampling frequency and a scanning range from 50 m to 3500 m.

These two instruments were deployed on the stern side of the CTV (see Fig. 2). The vessel is 27 m long and 10 m wide. The CTV typically did one round trip per day, leaving the harbour at around 7 am and returning around 6 pm. During the day, it moved within the wind farm depending on a daily varying repair and maintenance schedule. Hence, wind velocity data within the wind farm was collected for nearly twelve hours per day. As the vessel returned to the harbour in the evening, the lidars continued to measure, allowing the collection of data outside the wind farm. The scanning lidar’s south was aligned with the bow of the vessel, and the wind profiler’s east direction was similarly aligned. Consequently, the rotational data from the wind profiler could be used to correct the wind direction measurements in Doppler Beam Swinging (DBS) mode.

The WindCube 100S repeated a constant cyclical scanning pattern throughout the measurement period, alternating between continuous vertical stare mode for 25 minutes, and DBS wind profiling mode for 5 minutes. In a previous study^33^, the fixed LOS scanning mode of the WindCube 100S was validated against sonic anemometers, confirming its applicability for turbulence analysis. The range gate length was set to 25 m, and subsequent range gates were programmed with an overlap of 15 m, providing a nominal vertical resolution of 10 m over a scanning distance ranging from 50 m to 2 km above the surface. In the vertical stare mode, the lidar emits a laser beam directly upward at a 90° elevation angle. This method is especially effective for capturing the vertical wind velocity component, as the lidar measures the along-beam velocity component only.

During the observation of the KHBs on 2023-02-22, between 12:35 and 13:00, the vessel remained stationary within the farm. The closest wind turbine was positioned only 37 m upstream of the vessel (see Fig. 2 right), indicating that the lidar also captured the flow characteristics within the near wake region of that turbine. Although the study of near wake flows is an important topic in wind energy research^34,35^, it falls outside the main scope of this study.

## Data and methods

This study applies the notations used traditionally in boundary layer micrometeorology. Specifically, the wind velocity vector is decomposed into a mean component, denoted by an overbar, and a fluctuating component with zero mean value, denoted by a prime. For instance, the vertical velocity component can be decomposed as $w = \overline{w} + w^{\prime }$, which is assumed to be parallel to the vertical upward axis *z*. The data analysis takes place within the framework of boundary layer atmospheric flow over flat topography and time scales shorter than 30 minutes.

### Initial data processing and flow statistics

In the data pre-processing, all lidar data with a CNR below the threshold were dismissed. The threshold for WindCube V2 data was set at -24 dB and for the WindCube 100S at -27 dB. These threshold values are recommended by the manufacturer of the lidars^36^. Previous comparisons with ultrasonic anemometers have demonstrated that this threshold is sufficient in most cases^33^. Despite the potential of more advanced methods such as cluster analysis of CNR data to rescue data with lower CNR^37–40^, this approach did not significantly improve the dataset and was therefore not implemented.

Integral and spectral turbulence statistics were estimated after linearly detrending the velocity data^41^. While linear detrending is not always the optimal solution, it is often the best available approach to avoid data over-processing. Non-linear detrending can be controversial and often avoided as it may filter out large-scale fluctuations that could be of significant interest to the study of KHBs. The power spectral density (PSD) estimates were computed using Thomson’s multi-taper method^42^ with a time-half bandwidth product of 4, a value typically recommended in the literature^43^. Finally, a time-frequency analysis was conducted using the Evolutionary Power Spectral Density (EPSD) approach^44^. Further details on the EPSD method can be found in the Appendix. The EPSD method allows for the analysis of the spectral characteristics of the turbulence as they evolve, providing a dynamic view of the non-stationary processes that may be associated with the formation and dissipation of KHBs.

To contextualize the occurrence of the KHB, the atmospheric conditions before and after the event were studied by using wind speed and temperature data from the Norwegian hindcast archive NORA3. NORA3 is based on the HARMONIE-AROME numerical weather prediction model, which provides a hindcast of atmospheric data with a spatial resolution of 3 km and a temporal resolution of 1 hour^45^. Although NORA3 is not formally defined as a reanalysis product, it employs data assimilation of air temperature and relative humidity at 2 m above the surface.

To further understand the conditions leading to the KHB formation, the local bulk Richardson number $Ri_b(z)$ was computed at heights between 620 and 680 m above the surface as

1 $$\begin{aligned} Ri_b(z)&= \dfrac{g \cdot \Delta \overline{\theta } \cdot \Delta z}{\overline{\theta } (\Delta \overline{u})^2} \end{aligned}$$

where *g* is the gravitational acceleration, $\Delta \overline{\theta }$ is the mean potential temperature difference, $\Delta z$ is the vertical distance, $\overline{\theta }$ is the average potential temperature between the two height levels, and $\Delta \overline{u}$ is the corresponding horizontal wind speed difference. The potential air temperature ($\theta$) was calculated using temperature and pressure from NORA3. Even though the formation of turbulence and KHBs depends on local atmospheric gradients, we use the local bulk Richardson number, which incorporates the local gradients to approximate turbulent fluxes. Furthermore, the mean potential temperature profile from the NORA3 database serves as a necessary input for computing these gradients. All instances of $Ri_b$ exceeding the critical value of 1 are standardized to $Ri_b = 1$ for simplicity. The adjustment accounts for possible large uncertainties in the $Ri_b$ estimates or strongly stratified conditions. In this study, $Ri_b(z)$ was estimated using first the NORA3 data alone and then by combining the mean wind speed profile obtained from the WindCube 100S in DBS mode with the mean potential temperature profiles from NORA3 for an enhanced vertical spatial resolution.

### Flow stationarity assessment

As KHBs are transient events, the flow characteristics were calculated over carefully selected time intervals of less than 10 minutes, during which the assumption of flow stationarity is reasonably valid. Flow stationarity can be assessed using non-parametric tests, such as the reverse arrangement test^46^, or parametric tests, typically focusing on statistical moments^47^. We used a parametric test to study the stationarity of the velocity data recorded at 650 m above the surface. We study stationarity as *m*-order stationarity, where *m* represents the order of the statistical moment under consideration. For wind velocity data, first-order stationarity tests typically focus on the mean estimate and require knowledge of the instantaneous horizontal velocity components. Second-order stationarity tests can be applied to all velocity components to assess whether the variance or covariance remains relatively constant over time. In our study, we are interested in the turbulence statistics of the vertical component. Therefore, we concentrated on evaluating the second-order stationarity via its standard deviation. The time series was divided into three shorter segments: ’before KHB’ (12:35–12:44), ’during KHB’ (12:47–12:56), and ’after KHB’ (12:56–13:00). Second-order stationarity was evaluated by estimating the maximum absolute relative error between a moving standard deviation (*instantaneous* estimate, $\sigma _w(t)$) and the standard deviation of the entire segment (*static* estimate, $\sigma _w$):

2 $$\begin{aligned} \epsilon&= \text {max}\left( \left| 1 - \frac{\sigma _w(t)}{\sigma _w}\right| \right) \end{aligned}$$

We employed a 3-minute moving window and set a threshold of $\epsilon _{\text {max}} = 40\%$ for the relative error between *instantaneous* and *static* standard deviations. This threshold is empirically robust for identifying non-stationary flow in the atmospheric boundary layer^48^. For all segments, the condition $\epsilon < \epsilon _{\text {max}}$ was satisfied, confirming stationary fluctuations within each segment.

### Frequency-dependant noise treatment

In this study, lidar data at frequencies above 0.2 Hz are influenced by several factors: (1) spatial averaging due to the range-gate length of 25 m, leading to an underestimation of signal variance; (2) motion-induced velocities, which can lead to an overestimation of signal variance; and (3) contamination of the vertical velocity component by the projection of horizontal velocity components, when the lidar beam is not aligned in a precisely vertical orientation, due to the roll and pitch of the vessel.

We propose a new filtering function to partially reduce noise in vertical velocity fluctuations measured by the WindCube 100S lidar, where noise arises primarily from instrumental limitations and vessel motion. The method relies on a visually determined threshold frequency ($f_{\text {thresh}}$), identified as the point where the PSD of the vertical velocity fluctuations flattens, marking a transition to noise-dominated behaviour. More specifically, we identify the frequency at which the slope of the PSD deviates from the expected -5/3 power law and transitions to a noise-dominated regime. The filtering process consists of the following steps:

First, the PSD of the signal, $S_w(f)$, is calculated and the slope of the PSD above $f_{\text {thresh}}$ is estimated. The PSD in this region is modelled using a non-linear regression of the form:

3 $$\begin{aligned} S_w(f)&= a f^{-b}, \end{aligned}$$

where *a* is a scaling coefficient and *b* is the estimated spectral slope. Next, a spectral filter is applied in the frequency domain. The filter response, *H*(*f*), is defined as:

4 $$\begin{aligned} H(f) = {\left\{ \begin{array}{ll} 1, & \text {if } f \le f_{\text {thresh}}, \\ \left( \frac{f}{f_{\text {thresh}}} \right) ^{b/2} \cdot \left( \frac{f}{f_{\text {thresh}}} \right) ^{-5/6}, & \text {if } |f| > f_{\text {thresh}}, \end{array}\right. } \end{aligned}$$

where the first term flattens the PSD above $f_{\text {thresh}}$, and the second term enforces the − 5/3 spectral slope characteristic of the inertial subrange in turbulence.

The filter is applied to the amplitude spectrum of the signal while preserving its phase spectrum to maintain the temporal coherence of the filtered time series. The filtered time series is then obtained by applying the inverse Fourier transform. This approach reduces noise in the high-frequency range while preserving low-frequency data.

**Fig. 3:**
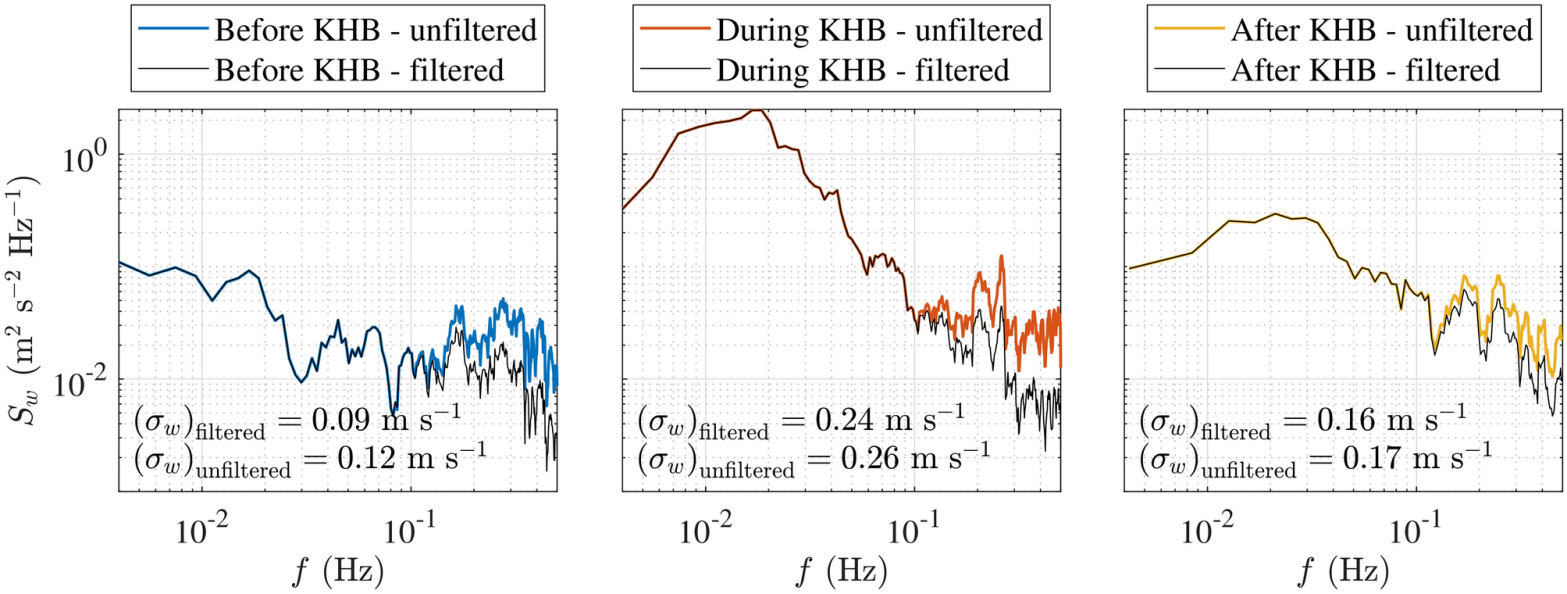
Power spectral density of the vertical velocity component at 650 m above the surface, before (left panel), during (middle panel) and after (right panel) the KHB observation, with and without filter.

Figure 3 visualises the PSD of the velocity fluctuations with and without filtering, before (left panel), during (middle panel) and after (right panel) the KHB event at the height of 650 m above the surface. Although the figure shows relatively small differences in the standard deviation of the vertical component, this standard deviation is typically underestimated due to the spatial averaging.

### Motion correction

The WindCube 100 in DBS mode emits a series of pulsed laser beams at a fixed elevation angle ($\phi =75^\circ$) across four azimuth angles ($\alpha$) of $0^\circ$, $90^\circ$, $180^\circ$, and $270^\circ$. The WindCube V2 also runs a 5-beam DBS, where the fifth beam is emitted at an elevation angle of $90^\circ$. The LOS velocity, denoted as $v_r$, recorded by the lidar for a specific half cone opening angle ($\theta = 90^{\circ } - \phi$) and azimuth angle $\alpha$, is related to the wind velocity vector ${\mathbf{u}} = (u, v, w)$ as

5 $$\begin{aligned} v_r = u\, \cos \alpha \, \sin \theta \, +\, v\,\sin \alpha \,\sin \theta \, + w\, \cos \theta . \end{aligned}$$

By solving

6 $$\begin{aligned} {{\mathbf{v}}_{\mathbf{r}}} = {\mathbf {N}} {\mathbf{u}}, \end{aligned}$$

the wind velocity can be reconstructed. In case of WindCube V2 with 5 beams

7 $$\begin{aligned} {{\mathbf{v}}_{\mathbf{r}}} = \begin{pmatrix} v_r(\alpha = 0^{\circ }) \\ v_r(\alpha = 90^{\circ }) \\ v_r(\alpha = 180^{\circ }) \\ v_r(\alpha = 270^{\circ }) \\ v_r (\phi = 90^{\circ }) \end{pmatrix},\quad {\mathbf {N}} = \begin{pmatrix} \sin \theta & \quad 0 & \quad \cos \theta \\ 0 & \quad \sin \theta & \quad \cos \theta \\ -\sin \theta & 0 & \quad \cos \theta \\ 0 & \quad -\sin \theta & \quad \cos \theta \\ 0 & \quad 0 & \quad 1 \\ \end{pmatrix}. \end{aligned}$$

In the presence of motion, Eq. 6 is modified to

8 $$\begin{aligned} {{\mathbf{v}}_{\mathbf{r}}} = {\mathbf {N}} {\mathbf {R}} ( {\mathbf{u}} + {{\mathbf {u}}_{\mathbf{T}}}), \end{aligned}$$

${{\mathbf{u}}_{\mathbf{T}}}$ represents the translational motion vector and **R** represents the rotation matrix. **R** is calculated as

9 $$\begin{aligned} {\mathbf {R}} = {{\mathbf {R}}_{\mathbf{x}}} {{\mathbf {R}}_{\mathbf{y}}} {{\mathbf {R}}_{\mathbf{z}}}, \end{aligned}$$

where

10 $$\begin{aligned} {{\mathbf{R}}_{\mathbf{x}}} = \begin{pmatrix} 1 & \quad 0 & \quad 0 \\ 0 & \quad \cos \beta & \quad \sin \beta \\ 0 & \quad - \sin \beta & \quad \cos \beta \\ \end{pmatrix},\quad {{\mathbf{R}}_{\mathbf{y}}} = \begin{pmatrix} \cos \varphi & \quad 0 & \quad -\sin \varphi \\ 0 & \quad 1 & \quad 0 \\ \sin \varphi & \quad 0 & \quad \cos \varphi \\ \end{pmatrix},\quad {{\mathbf{R}}_{\mathbf{z}}}= \begin{pmatrix} \cos \psi & \quad \sin \psi & \quad 0 \\ -\sin \psi & \quad \cos \psi & \quad 0 \\ 0 & \quad 0 & \quad 1 \\ \end{pmatrix}, \end{aligned}$$

corresponding to the roll ($\beta$), pitch ($\varphi$), and yaw ($\psi$) angles respectively. By solving the Eq. 8, the corrected wind vectors are retrieved^30^. This motion correction technique can be applied to both the wind profiler lidar (WindCube V2) and the scanning lidar (WindCube 100S) operating in DBS profiling mode given that the roll, pitch, yaw angles and translational velocities are known at each time step.

When the WindCube 100S operates in vertical stare mode, it employs only a single beam, resulting in only one equation to solve for three unknowns. Consequently, this motion correction method cannot be applied, as there is insufficient information for fully correcting the vertical velocity component (*w*) in this configuration. Moreover, since the scanning lidar is installed on the vessel without a real-time mechanical motion compensation system such as other studies^49^, slight variations in the orientation of the lidar beams are observed. Therefore, in vertical staring mode, the LOS velocity measured by the lidar is not an exact measure of the vertical wind velocity component. The LOS velocity includes contributions from both horizontal velocity components *u* and *v*, depending on the pitch and roll of the vessel.

To correct the static tilt of the vertical beam, we have developed a new method based on the double rotation technique, typically used for correcting static tilt angles in sonic anemometers^50^. Our correction method is innovative as it combines the double rotation technique with data from two lidar systems.

The corrected vertical velocity component, $w_c$, is given by:

11 $$\begin{aligned} \overline{w}_c = -\overline{u} \sin \left( \theta \right) + \overline{w}_{uncor} \cos \left( \theta \right) , \end{aligned}$$

the overbar denotes the temporal average; $\theta$ is the tilt angle between the true vertical axis and the axis along which the uncorrected vertical velocity component $w_{uncor}$ is measured.

In this study, the tilt angle for the WindCube 100S is unknown. We solve Eq. 11 for $\theta$ at heights between 200 and 290 m using the motion-corrected $w_c$ and $\overline{u}$ from the WindCube V2, and $w_{uncor}$ from the WindCube 100S. Using data from 12:56 to 13:00, during which all sensors provided high-quality data, we identified an average tilt angle of 2.7°, consistent with initial visual observation at the beginning of the campaign. The corrected profile of the vertical velocity component was then estimated a second time using Eq. 11. For this step, $\overline{u}$ was the mean wind speed estimated by the WindCube 100S to extend the analysis to altitudes beyond the range of the WindCube V2.

## Results

Numerous KHB events were observed at altitudes ranging from a few hundred meters to above 1 km during the measurement campaign in 2022 and 2023. Two additional KHB observations are presented in the Appendix. Hereinafter, we only focus on the KHB observed on 2023-02-22, between 12:35 and 13:30 (UTC). The in-situ measurements, as well as SCADA data from the wind turbines surrounding the vessel, indicate that the wind was blowing from the southeast at the time of this KHB event.

**Fig. 4:**
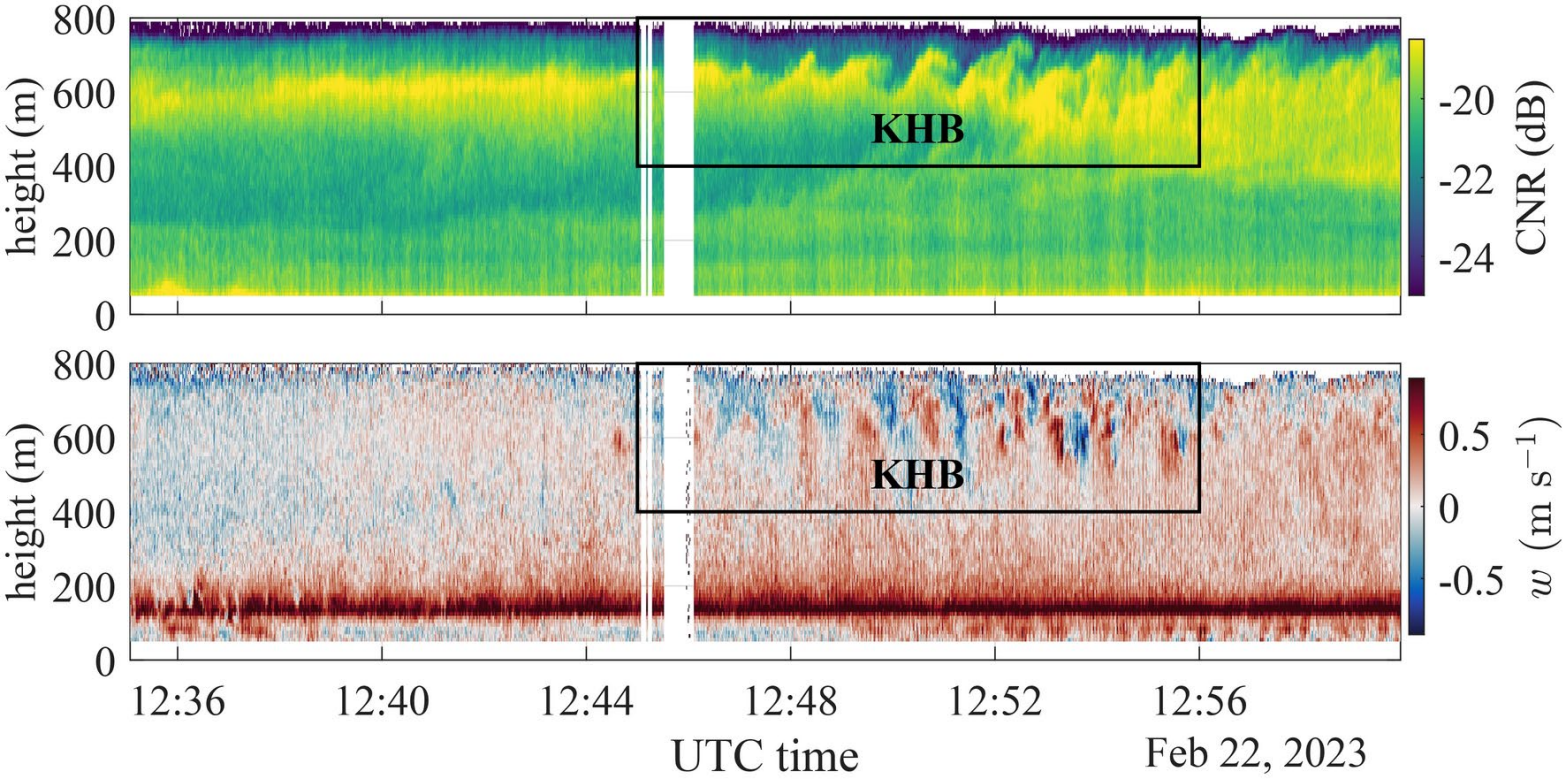
Time series of the CNR (top panel) and vertical velocity component (bottom panel) recorded by the WindCube 100S in vertical stare mode from 12:35:04 to 12:59:56 on 2023-02-22. The box indicates the time during which the KHB event occurred. The vessel was not moving during the observation period.

Figure 4 shows the CNR and vertical velocity measured by the scanning lidar (WindCube 100S) at 1 Hz resolution during vertical stare observations from 12:35 to 13:00, spanning the KHB event. The lidar measurement range extended from 50 m to approximately 800 m above the surface, reaching the top of the ABL. Above, in the free atmosphere, the aerosol concentration is largely reduced and can thus no longer provide sufficient backscatter for reliable lidar measurements. The top panel presents time-height contour plots of the CNR. High values, shown in yellow, correspond to enhanced aerosol concentrations causing increased backscatter and show in this case a multi-layer structure of the ABL, with a distinct maximum concentration at around 600 m. Indications of a secondary maximum can be seen close to the ground. The bottom panel shows the corresponding wind velocity fluctuations. Positive values, marked in red, indicate motion away from the lidar, i.e., upward motion, negative values in blue correspond to downward motion. The dark red line structure between 150 m and 200 m, indicating upward directed vertical velocities with a maximum speed of around 1 m s^−1^, corresponds to a near wake of the wind turbine located in the vicinity of the vessel.

The time series of CNR profiles in Fig. 4 highlights significant KHB structures between 12:46 and 12:56 at heights of 550 to 750 m. It visualises billows at all stages of development, beginning with small wave disturbances that grow in amplitude, overturn, and eventually break. In the vertical velocity field, these billows also appear as distinct alternations between upward and downward motion. The bright CNR band around 600 m, visible before the formation of KHBs, indicates aerosol accumulation below the capping inversion of the ABL. Such inversions are typically associated with substantial wind shear, which is necessary for initiating the observed KHBs. The KHB structure is advected through the lidar beam over a time period of approximately 11 min, and the KHB event causes distinct mixing, visualised by the broadening and reduction in brightness of the CNR maximum. At 13:00, this mixing reaches down to nearly 350 m.

**Fig. 5:**
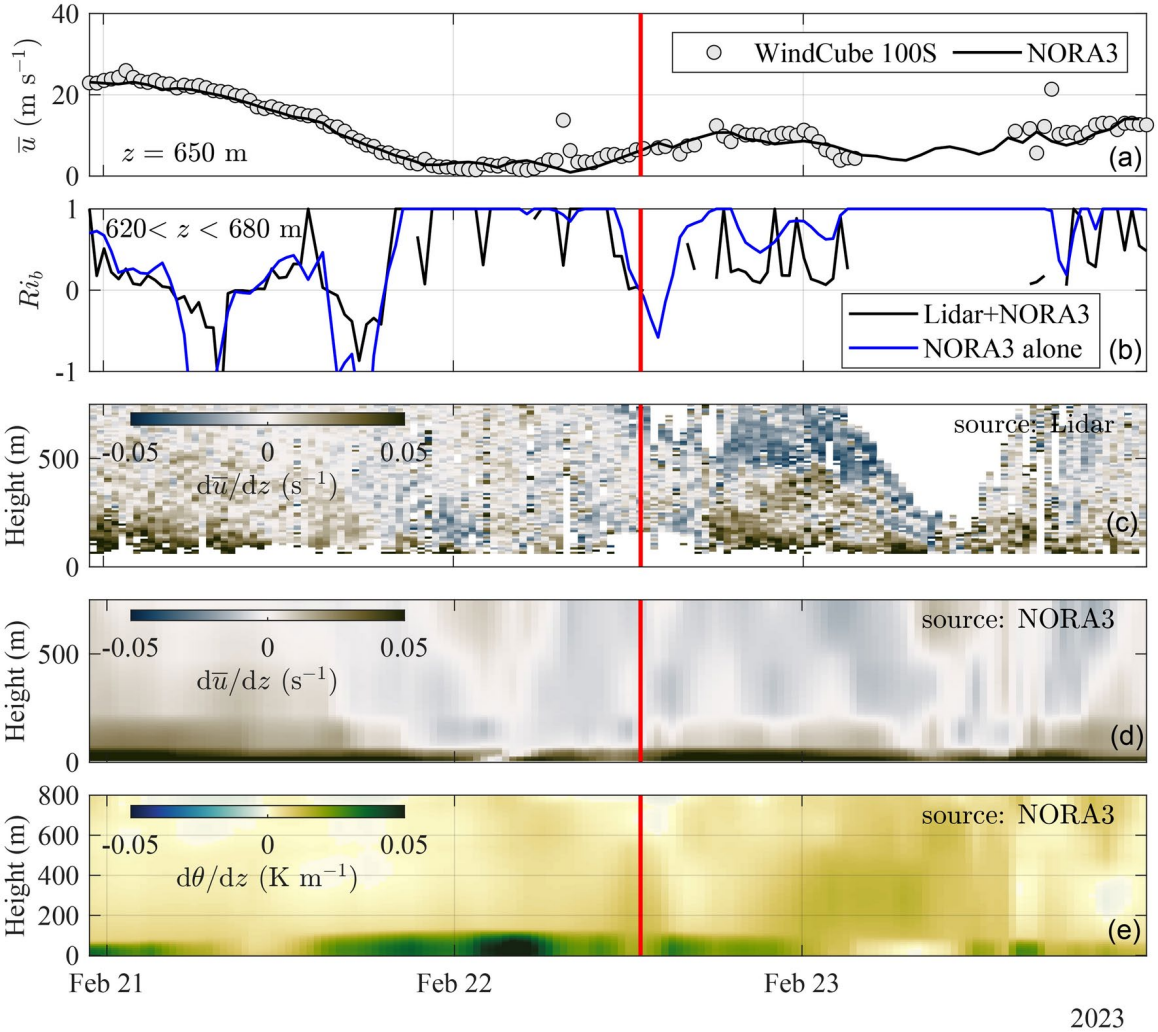
Hourly time series showing mean wind speed at 650 m from NORA3 and the scanning Doppler wind lidar (**a**), local bulk Richardson number (**b**), wind speed gradient estimated using the scanning lidar in DBS mode (**c**) and NORA3 data (**d**), and vertical gradient of potential temperature from NORA3 data (**e**). Red lines indicate the timing of the KHB event.

Figure 5 provides an overview of the broader atmospheric conditions over four days around (before and after) the observation of KHBs on 2023-02-22. The wind speed at an altitude of 650 m, shown in panel (a) and measured by the Doppler scanning wind lidar (WindCube 100S) in DBS mode, declines markedly during the 48 hours preceding the KHB event. It decreases from peak values of approximately 30 m s^−1^ on 2023-02-20 to 2 m s^−1^ on the morning of 2023-02-22. This trend is consistent with wind speed data from the NORA3 hindcast database. The local bulk Richardson number ($Ri_b$), estimated using either NORA3 data alone or a combination of lidar and NORA3 data, shows positive values both below the critical threshold of 0.25^51^, which is a necessary condition for Kelvin–Helmholtz instability^13,52^. Following the KHB event, $Ri_b$ briefly dips into negative values before returning to positive. This indicates enhanced mixing potentially triggered by the KHB event. The wind speed gradient profile, calculated from lidar and NORA3 data and shown in Fig. 5, exhibits negative shear a few hours prior to the KHB event. Meanwhile, the gradient of potential temperature remains positive, indicating a statically stable atmosphere. After the KHBs, the atmosphere remains statically stable, indicated by a consistent positive potential temperature gradient, while a negative vertical wind speed gradient persists. Despite NORA3’s lower vertical resolution, both NORA3 and the lidar data capture similar positive and negative shear profiles. Discrepancies between lidar and model data become more apparent after the KHB event. Specifically, the lidar data indicate that a maximum of local wind speed, not captured by NORA3, is reached by the end of 2023-02-22. It is important to note that Fig. 5 does not attempt to detect or directly identify the KHBs. Instead, it offers a contextual background, which can be useful for future studies aiming to reproduce this event through simulations or further analysis.

**Fig. 6:**
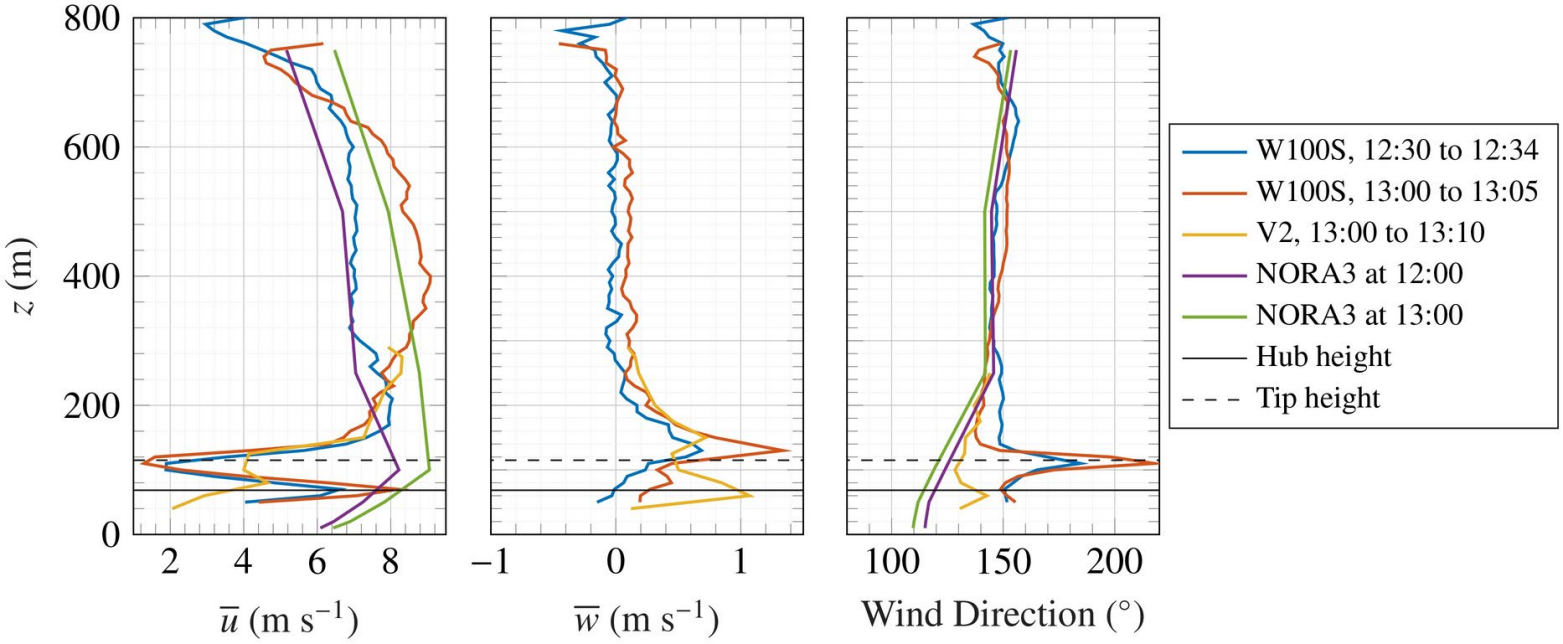
Mean horizontal (left panel) and vertical (middle panel) wind speed profiles, along with the mean wind direction (right panel) from the WindCube 100S before (blue) and after (orange) KHB. Additionally, data from the WindCube V2 after KHB (yellow), NORA3 before (purple) and after (green) KHBs are shown. Hub height is shown by the black solid line, while tip height is shown by the black dashed line.

Figure 6 presents the vertical profiles of the horizontal mean wind speed (left panel), vertical mean wind speed (middle panel), and mean wind direction (right panel). The hub height and tip height of the wind turbine are marked with solid and dashed lines, respectively. The left panel of Fig. 6 confirms the existence of a negative wind shear before and after the observation of the KHB, with a decrease from roughly 7 m s^−1^ at 600 m to 4 m s^−1^ at 800 m, leading to a wind shear of approximately − 0.02 m s^−1^ per meter. This is consistent with large-eddy simulations of KHB by Jiang^53^, who estimated a negative wind shear of − 0.03 m s^−1^ per meter for KHBs occurring 300 m above the surface.

Additionally, multiple inflexion points are visible in the pre-KHB wind speed profile (12:30 to 12:34), especially around 600 m and 750 m, which are essential for the development of Kelvin–Helmholtz instability, as indicated by Blumen et al.^13^. Although wind speed data from NORA3 show larger deviations from the WindCube 100S lidar data, such discrepancies are expected, as numerical weather prediction models often struggle to represent stable flow conditions accurately^54^. It should be mentioned that the horizontal mean wind speed profiles were obtained using the WindCube 100S during a five-minute DBS scan both pre- and post-KHB, alongside NORA3 data and the 10-minute profile from the WindCube V2 at the nearest available timestamp. Given the lidar’s position in the near-wake area of a wind turbine, the flow in this region is notably disturbed and heterogeneous. Therefore, the fundamental assumption of horizontal homogeneity-necessary for accurate wind profiling in DBS mode-may be violated at heights below 200 m, increasing measurement uncertainty. As the primary focus of this work is the study of KHBs, only wind velocity data above 200 m will be discussed henceforth. 

To study the vertical mean wind speed, data from both the WindCube V2 and the WindCube 100S were utilised, as shown in the middle panel of Fig. 6. The two instruments display consistent results at heights above 200 m. The positive peak in *w* around turbine tip height indicates the influence of the turbines’ structure and rotation on the local flow field, but this effect is small above 200 m. Observations show that the vertical velocity component remains relatively constant before and after the KHB event at altitudes between 200 and 600 m. However, the horizontal velocity increases compared with pre-KHB levels at the same altitude range. In the right panel of Fig. 6, the wind direction measured by the scanning lidar WindCube 100S is superposed to the wind direction from NORA3 and the profiler lidar WindCube V2. Wind direction data from NORA3 agrees well with in-situ measurement data at heights above 200 m, which appear to be unaffected by the near-wake of the turbine.

**Fig. 7:**
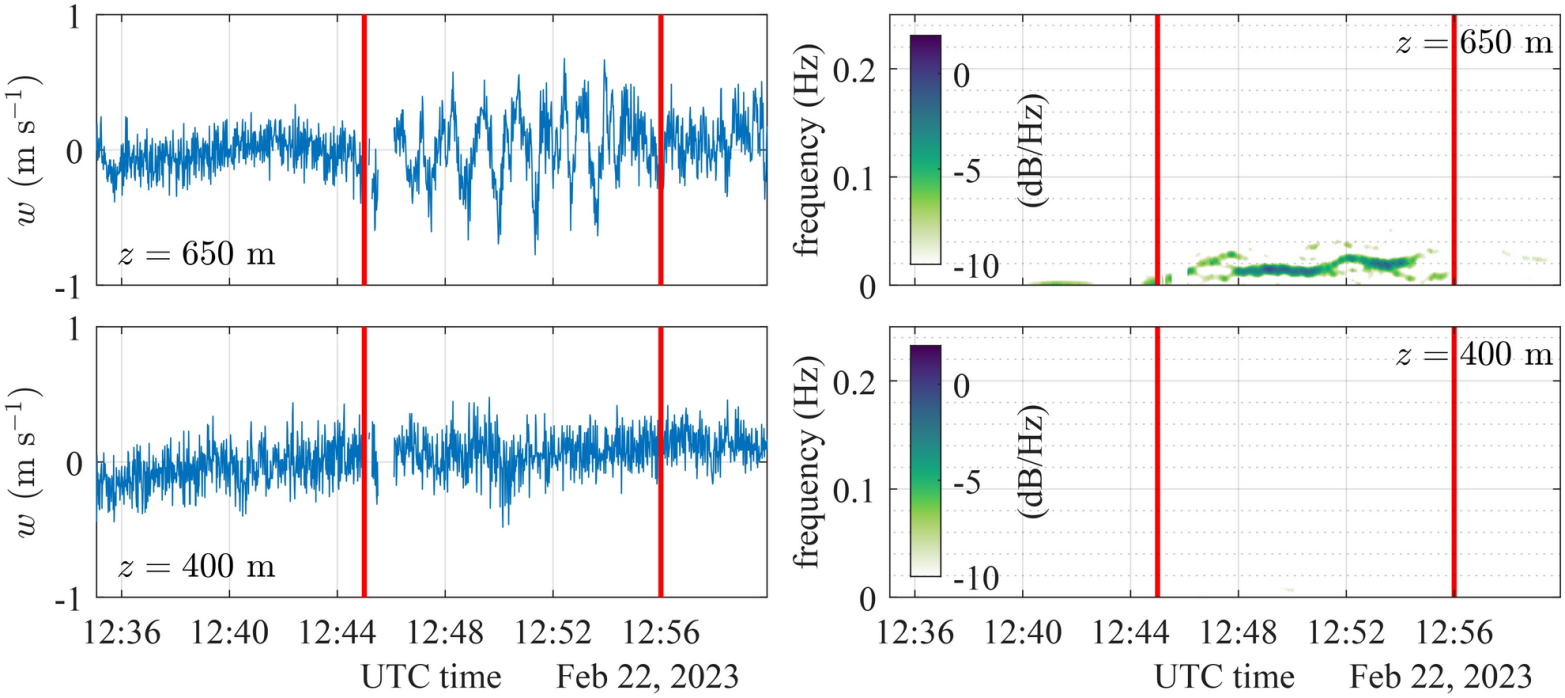
Vertical velocity recorded and the corresponding evolutionary power spectral density at 650 m (top panels) and 400 m (bottom panels) above the surface. A strong periodic pattern emerges between 12:45 and 12:56, during which the KHBs were observed.

A time-frequency analysis was conducted on the vertical velocity data collected by the WindCube 100S in vertical stare mode at 400 m and 650 m above the lidar before, during, and after the KHB observation (Fig. 7). EPSD exhibits a clear periodic pattern at a frequency of approximately 0.019 Hz, i.e., a period of 55 s. This reflects the periodic fluctuations of the vertical velocity related to the KHBs. This periodic pattern is transient, appearing approximately at 12:45 and disappearing near 12:56, consistent with the KHBs observed in the CNR data in Fig. 4. At 400 m, however, such a pattern is no longer visible in the EPSD around 12:50, indicating that the influence of KHBs remains well above the turbine tip height. Results show the efficacy of EPSD in identifying the presence of KHBs, particularly in assessing whether their signatures can be identified at lower altitudes, where they are not clearly visible in the time series of the CNR.

**Fig. 8:**
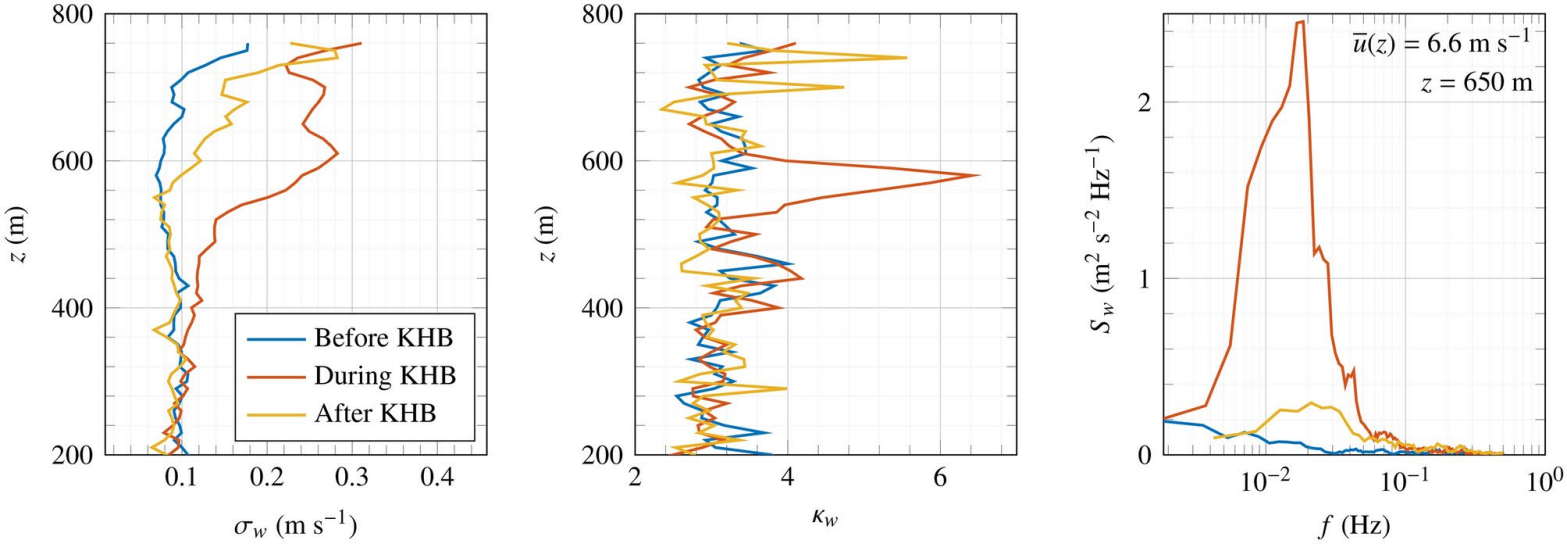
Profile of the standard deviation (left panel) and the kurtosis (mid panel) of the vertical velocity component, and the power spectral density estimate of the vertical component at 650 m above the surface (right panel), before, during and after the occurrence of the KHBs.

Figure 8 shows the profiles of the standard deviation $\sigma _w$ and kurtosis ($\kappa _w$) of the vertical velocity component, as well as its PSD at 650 m above the surface. The data is segmented into three distinct phases relative to the KHBs occurrence: ’before KHB’ (12:35-12:44), ’during KHB’ (12:47-12:56), and ’after KHB’ (12:56-13:00). While the standard deviation of vertical wind before and after the event is similar, it substantially increases during the KHBs at heights between 500 m and 700 m above the surface. More specifically, at a height of 650 m, $\sigma _w$ is 2.3 times greater during the KHB than before. This increase in turbulence fluctuations is also visible in the PSD of the vertical velocity component ($S_w$).

The kurtosis profiles indicate a marked increase at approximately 575 m, with $\kappa _w > 6$, exceeding the standard kurtosis value of $\kappa _w = 3$ associated with Gaussian fluctuations. Previous studies, including those by Blumen et al.^13^ based on sonic anemometer measurements, and Werne and Fritts^55^ by direct numerical simulations of Kelvin–Helmholtz instability, have documented substantial increases in the kurtosis of the vertical velocity component near the edges of KHBs. Blumen et al.^13^ noted that a marked increase in $\kappa _w$ signifies intensified intermittency and augmented entrainment. In our study, local enhancements of $\kappa _w$ suggest that KHBs did not extend spatially below 550 m. This finding is consistent with the EPSD plots in Fig. 7. Outside the areas affected by KHBs, the profiles of $\kappa _w$ and $\sigma _w$ exhibit most of the time characteristics of homogeneous and Gaussian turbulence, consistent with the decomposition of the 25-min time series into three shorter stationary segments.

Additional scans conducted before (12:20–12:30) and after (13:05-13:10) the observation of the KHB did not show any distinct peaks in the vertical profiles of $\kappa _w$, suggesting that the KHB recorded between 12:47 and 12:56 was relatively short-lived. However, a local maximum in the CNR was still visible in the scan from 13:05 to 13:30, although it lacked the structured characteristic of KHBs. The PSD of the vertical velocity component reveals a significant increase at frequencies between ${4\times 10^{-3}}$ Hz and ${4\times 10^{-2}}$ Hz. In particular, the spectral peak at approximately ${2\times 10^{-2}}$ Hz is nearly 30 times greater during KHB than before. This pronounced rise in the $S_w$ spectrum is relatively short-lived. After the KHB event, $S_w$ values return to magnitudes similar to those observed prior to the event. Further observations and modelling are needed to understand the significance and generalisability of the observed dominant frequency within this spectral peak.

**Fig. 9:**
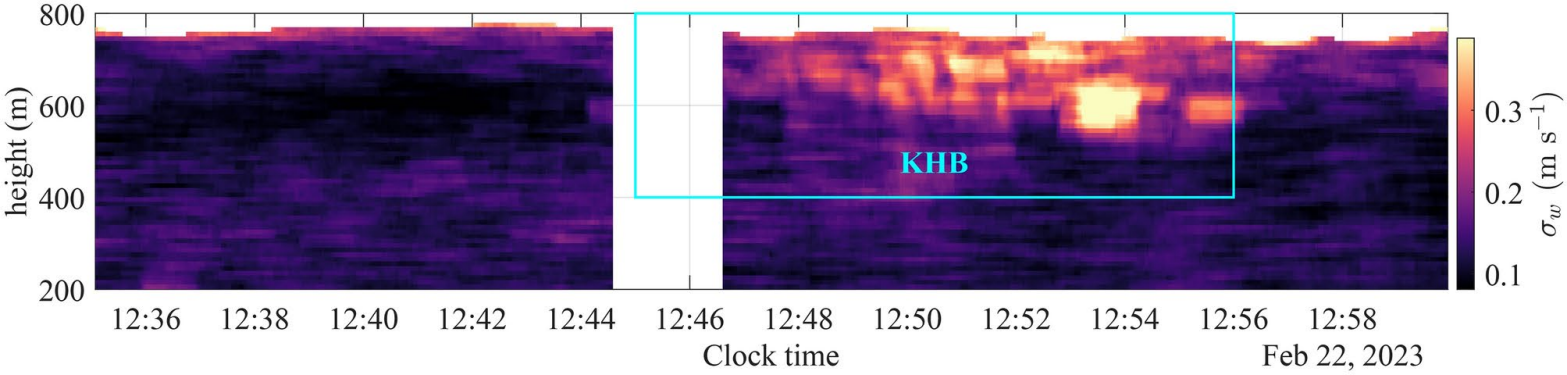
Profile of the instantaneous standard deviation of the vertical velocity component, estimated using a centred sliding window of 60 s. The cyan box corresponds to the period labelled ’During KHB’ in Fig. 8.

Using a sliding window function, Fig. 9 presents the ’instantaneous’ profiles of $\sigma _w$. While it is possible to estimate instantaneous profiles of kurtosis ($\kappa _w$), being a higher-order characteristic of turbulence, it requires a significantly longer averaging period than that needed for $\sigma _w$ to ensure reliable accuracy^56^. Therefore, this analysis focuses exclusively on the temporal variability of $\sigma _w$. Figure 9 confirms that the notable increase in $\sigma _w$, observed in Fig. 8, is a transient phenomenon confined to altitudes above 550 m. Consistent with the findings of Blumen et al.^13^, the surge in $\sigma _w$ exhibits intermittency. However, the subsequent decrease occurs marginally slower, resulting in a slightly elevated $\sigma _w$ post-KHB compared to its initial state.

## Conclusions

This study presents observations of Kelvin–Helmholtz Billows (KHBs) captured by a scanning Doppler wind lidar mounted on a vessel deployed within an offshore wind farm in Denmark in 2023. The lidar operated in vertical stare scanning mode, allowing for the characterisation of the vertical turbulence structure up to the top of the atmospheric boundary layer with a spatial resolution of 10 m and a temporal resolution of 1 Hz. Mean wind speed profiles were collected every 25 minutes using the same instrument in Doppler Beam Swinging (DBS) profiling mode, and every 10 minutes using a WindCube V2 lidar profiler with a maximal range of 300 m.

To the authors’ knowledge, this is the first instance of such high-accuracy measurements being obtained from a vertically-pointing scanning wind lidar. Furthermore, we demonstrate that slant profiles of the carrier-to-noise ratio (CNR) can complement lidar velocity data for studying coherent structures in the atmospheric boundary layer. This study quantifies the spatio-temporal characteristics of the KHBs, supplementing previous in-situ observations. The results provide the following key findings:

- The selected lidar setup allows the detection of KHB events in clear sky conditions with high spatial and temporal resolution. The chosen case study was characterized by statically stable conditions and a moderate wind speed of around 8 m s^−1^. The mean wind direction remained nearly constant during the observation period. A strong negative shear was observed above 600 m, the altitude above which the KHBs formed.
- The formation of KHBs is associated with a clear periodic vertical velocity fluctuation pattern, with a period of approximately 55 s, consistent with previous observations of KHBs in the atmospheric boundary layer. A time-frequency analysis based on the evolutionary power spectral density successfully identified altitudes at which the KHBs affected ambient turbulence. This shows the potential of this approach for automatic KHB identification. Time-varying profiles of the standard deviation of the vertical velocity component were also able to pinpoint the time at which the KHB forms and dissipates.
- The formation of KHBs is associated with a substantial increase in vertical turbulence. More specifically, the local vertical turbulence intensity during the KHB is twice that observed before the event. The power spectral density (PSD) of the vertical velocity component showed an even greater increase in the frequency range below 0.1 Hz.
- Profiles of the kurtosis $\kappa _w$ of the vertical velocity component exhibited a surge near the edges of the billows, consistent with prior observations and direct numerical simulations available in the literature. In our study, we also observed a local increase in $\kappa _w$ a few minutes before and after the KHB became visually detectable through the CNR.

This case study demonstrates the potential of operating a ship-borne scanning Doppler wind lidar system in a combination of vertical stare and DBS modes to study the marine atmospheric boundary layer. The lidar observations, complemented by high-resolution NORA3 hindcast data, form a unique dataset for future research. With numerous samples collected between September 2022 and August 2023, this dataset is expected to provide detailed insights into the structure and dynamics of the marine atmospheric boundary layer both within and around a wind farm.

The results of this case study suggest that such measurements may also be highly relevant to the field of wind energy meteorology. The KHBs observed in this study (see also the Appendix) occur at altitudes where future airborne wind energy systems (AWES) are expected to operate, potentially impacting energy harvesting efficiency, flight stability, and the load and fatigue of these systems. As shown in the Appendix and by Jiang^57^, Kelvin–Helmholtz instabilities can develop at lower levels in the atmosphere or near the surface. We therefore hypothesize that the increased vertical turbulent mixing generated by KHBs could accelerate the recovery of wakes generated by individual turbines or entire wind farms under stable conditions. This hypothesis echoes the findings of Radunz et al. (2025)^58^, who, through simulations, noted the possible interaction between KHB and wind turbines. However, further study is needed to understand the underlying recovery mechanism and the extent to which KHB might accelerate wake recovery.

## Supplementary Information

Supplementary Information.

## Data Availability

Supporting data for Figs. 4, 7 and 8 namely the vertical velocity component and CNR recorded by the WindCube 100S in vertical stare mode at the time of the KHB observation, are available in the figshare repository: https://figshare.com/s/2cd5651f15307dcdd7fa. The WindCube 100S DBS wind velocity recorded at the time of the KHB observation supporting Figs. 5 and 6 is also available on the same figshare repository. The SRTM data V4, from Jarvis A., H.I. Reuter, A. Nelson, E. Guevara, 2008, provided by the International Centre for Tropical Agriculture CIAT, (https://srtm.csi.cgiar.org) supports Fig. 1. Supporting data for Fig. 5 on the temperature and mean wind speed data can be obtained from the Norwegian hindcast archive NORA3 (https://thredds.met.no/thredds/projects/nora3.html). WindCube V2 metadata are available in the figshare repository: https://doi.org/10.11583/DTU.22739729.v1. However, these data are currently being processed for another manuscript and will be available after its publication. SCADA data were acquired from RWE Group, and the corresponding data is protected by the company confidentiality policy.
