# Supplementary material for: Observation of Kelvin–Helmholtz billows in the marine atmospheric boundary layer by a ship-borne Doppler wind lidar
Source: Sci Rep. 2025 Feb 12;15:5245. doi: 10.1038/s41598-025-89554-4 (PMC11822104; doi:10.1038/s41598-025-89554-4)
Supplement: Supplementary file 1 — Supplementary Information. [file 41598_2025_89554_MOESM1_ESM.pdf]

A Evolutionary Power Spectral Density

We describe below the evolutionary Power Spectral Density (EPSD) with the notation from Hu et al. (2024)[1] at angular frequency ω and time t is given by the following set of equations:

$$S(\omega,t) = \int_0^{+\infty} W(\tau)\,|\tilde{x}(\omega,t-\tau)\tilde{x}^*(\omega,t-\tau)|\,d\tau \quad (1)$$

$$\tilde{x}(\omega,t) = \int_0^{+\infty} g(\tau)x(t-\tau)e^{i\omega(t-\tau)}d\tau \quad (2)$$

Where: $S(\omega,t)$ is the EPSD at angular frequency ω and time t and $W(\tau)$ is the smoothing window function; $x(t)$ is the original time-domain signal; $g(\tau)$ is the weighting function applied over time shifts τ. The complex conjugate is denoted by a subscript asterisk, z^*, where z is a complex number. The weighting function $g(\tau)$ is defined as:

$$g(\tau) = \begin{cases} \frac{1}{2\sqrt{h\pi}} & |\tau| \leq h \\ 0 & |\tau| > h \end{cases} \quad (3)$$

where h is the window size for the weighting function $g(\tau)$. The smoothing window $W(\tau)$ is defined as:

$$W(\tau) = \begin{cases} \frac{1}{\Gamma} & |\tau| \leq \frac{\Gamma}{2} \\ 0 & |\tau| > \frac{\Gamma}{2} \end{cases} \quad (4)$$

where Γ is the width of the smoothing window. In this study, we used $h = 60\Delta t$ and $\Gamma = 1.01\Delta t$ where Δt is the time step. These values are typically obtained by trial and error, depending on the temporal and frequency scale of the phenomenon observed.

B Additional KHB observations

Numerous additional KHBs have been observed at heights ranging from a few hundred metres to above 1 km above the surface during the Lollex campaign. Figure B.1 illustrates a short-lived KHB that also displays enhanced vertical turbulence fluctuations at a height of around 500 m above the surface. Figure B.2 depicts two KHBs at around 600 m and 1250 m above the surface. Assuming that vertical velocity variance σ_w is an appropriate proxy for turbulent kinetic energy, we can hypothesise that the increased values of σ_w also reflect increased turbulent mixing, as indicated by Sun et al.[2]. Therefore, the observations of the KHBs suggest that the billows facilitate vertical momentum entrainment at the top of the atmospheric boundary layer. The vertical spatial extent of KHBs increases further away from the surface, making their detailed observation and study by the scanning Doppler wind lidar more challenging at lower heights.

References

1. Hu, W., Yang, Q., Peng, L., Liu, L., Zhang, P., Li, S. & Wu, J. Non-stationary modeling and simulation of strong winds. *Heliyon* **10**, 15 (2024).
2. Sun, J., Mahrt, L., Nappo, C. & Lenschow, D. H. Wind and temperature oscillations generated by wave–turbulence interactions in the stably stratified boundary layer. *J. Atmospheric Sci.* **72**, 4, 1484–1503 (2015).

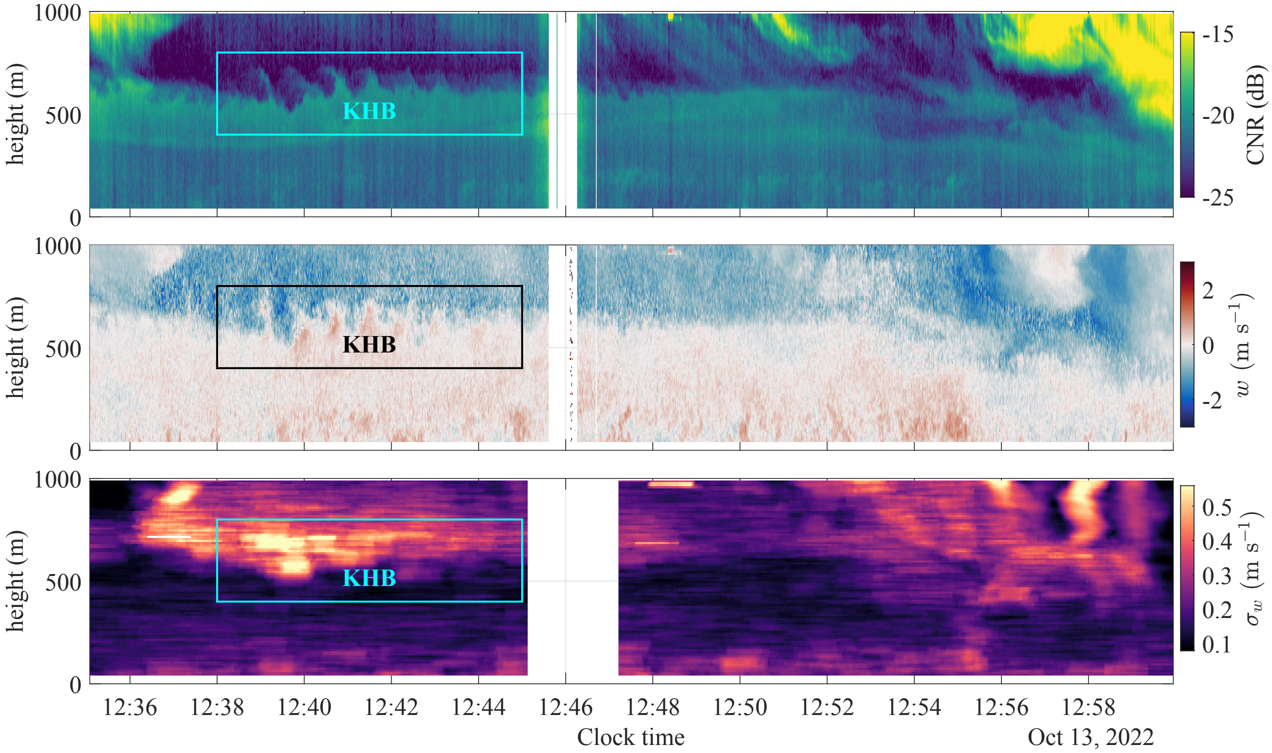

Figure B.1. Additional KHBs observed on 2022-10-13 between 12:38 and 12:45 at heights around 500m.

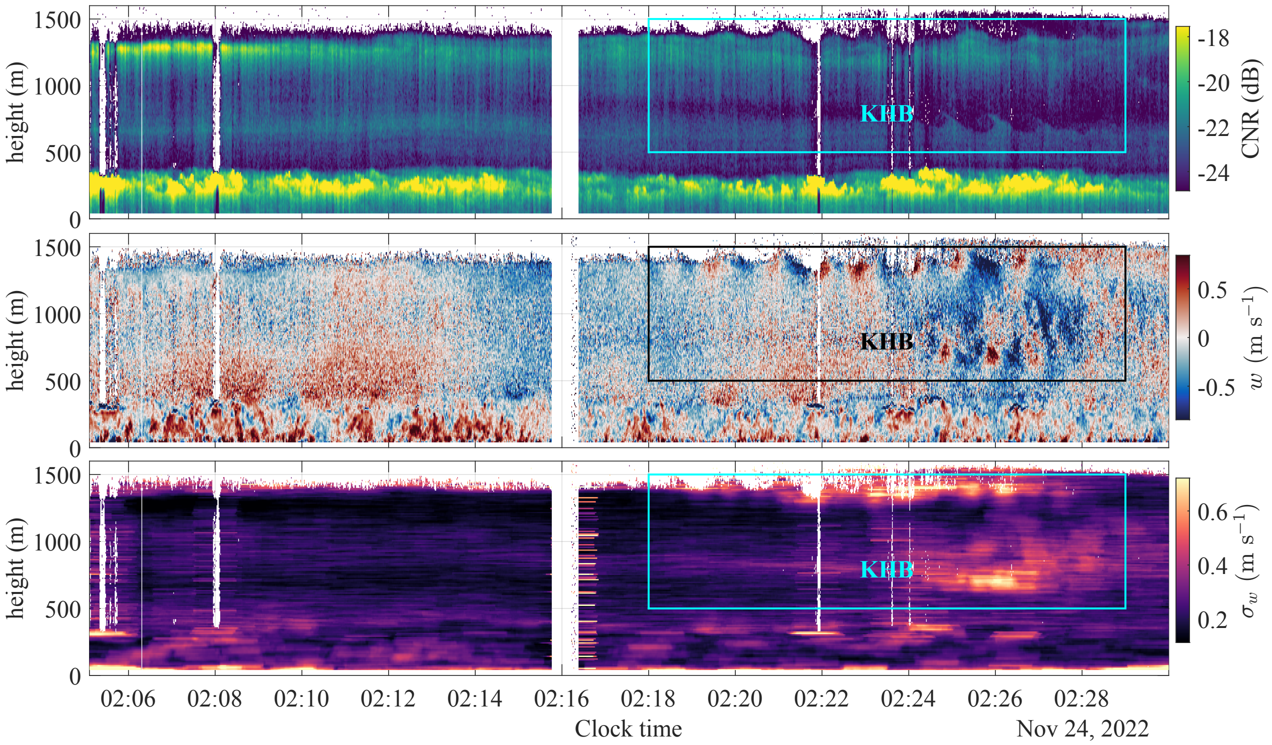

Figure B.2. Two KHBs observed on 2022-11-24 between 12:38 and 12:45 at heights around 600 m and 1250 m above the surface. The nocturnal boundary layer extends up to almost 400 m and the residual layer reaches up to 1500 m above the surface.
